# Delayed epistaxis after endoscopic transnasal pituitary tumor resection: clinical characteristics, risk factors, treatment and prevention

**DOI:** 10.1186/s12957-024-03428-z

**Published:** 2024-06-01

**Authors:** Chengzhi Mu, Zhenyu Song, Shengyuan Yu

**Affiliations:** 1 Department of Neurosurgery, Shandong Provincial Hospital Affiliated to Shandong First Medical University, 9677 Jingshi Road, Lixia District, Jinan, Shandong China; 2 Department of Neurosurgery, Linyi Traditional Chinese Medical Hospital, Linyi, Shandong China

**Keywords:** Endoscopic transnasal approach, Delayed epistaxis, Pituitary tumor resection

## Abstract

**Background:**

Delayed epistaxis after endoscopic transnasal pituitary tumor resection (ETPTR) is a critical complication, tending to cause aspiration or hemorrhagic shock. This study assessed clinical characteristics, risk factors, and provide treatment and prevention advice of this complication.

**Methods:**

This was a retrospective monocentric analysis of 862 patients who underwent ETPTR. Statistical analyses of clinical data revealed the incidence, sources and onset time of delayed epistaxis. Univariate analysis and binary logistic regression were used to identify risk factors.

**Results:**

The incidence of delayed epistaxis was 2.78% (24/862), with an average onset time of 20.71 ± 7.39 days. The bleeding sources were: posterior nasal septal artery branch of sphenopalatine artery (12/24), multiple inflammatory mucosae (8/24), sphenopalatine artery trunk (3/24) and sphenoid sinus bone (1/24). Univariate analysis and binary logistic regression analysis confirmed that hypertension, nasal septum deviation, chronic rhinosinusitis and growth hormone pituitary tumor subtype were independent risk factors for delayed epistaxis. Sex, age, history of diabetes, tumor size, tumor invasion and operation time were not associated with delayed epistaxis. All patients with delayed epistaxis were successfully managed through endoscopic transnasal hemostasis without recurrence.

**Conclusions:**

Delayed epistaxis after ETPTR tends to have specific onset periods and risk factors. Prevention of these characteristics may reduce the occurrence of delayed epistaxis. Endoscopic transnasal hemostasis is recommended as the preferred treatment for delayed epistaxis.

## Introduction

Endoscopic transnasal approach offers irreplaceable advantages over traditional microscopic transnasal approach, especially for large pituitary adenoma resection with suprasellar or parasellar extension [1]. However, with the expansion of endoscopic approach, damage to nasal structures is also inevitable. Compared to microscopic transnasal surgery, endoscopic transnasal surgery has a greater incidence of postoperative epistaxis, ranging from 0.2 to 4.3% [2, 3]. Epistaxis occurring 72 h or later after surgery was defined as delayed epistaxis. Delayed epistaxis, especially after discharge, can impose serious psychological stress on patients. Improper management may cause aspiration or hemorrhagic shock, threatening patient’s life [4]. Therefore, the prevention and treatment of delayed epistaxis should be taken seriously by endoscopic surgeons. This study aimed to explore the clinical features, risk factors, treatment and prevention of delayed epistaxis after endoscopic transsphenoidal pituitary tumor resection (ETPTR).

## Materials and methods

### General information

We retrospectively analyzed 862 cases of ETPTR performed at a single academic medical center from January 2017 to December 2021. Clinical data, including sex, age, hormone classification, past medical history, preoperative imaging, surgical approach and operation time, were collected and analyzed. For delayed epistaxis patients, the onset time, bleeding sources, and intraoperative nasal mucosa condition were also analyzed. All patients provided written informed consent to participate in this study, which was approved by ethics committee of Shandong provincial hospital affiliated to Shandong first medical university (SZRJJ: NO.2021).

### Endoscopic transnasal pituitary tumor resection

All patients were treated via a transsphenoidal approach and a nasoseptal rescue flap [5]. The procedure was as follows: the bilateral nasal cavity was rinsed with iodophor and nasal mucosa was convergent with adrenaline cotton. The more spacious side of the nasal cavity was selected for entry. The middle and superior turbinate were outfractured under direct visualization with an elevator, and the position of the opening of the sphenoid sinus was confirmed. Using an extended guarded needle-tip monopolar electric scalpel, a horizontal incision was made at the level of the sphenoid ostium superiorly approximately 1 cm from the skull base to preserve the olfactory filaments. This incision was extended 2 cm horizontally and turned 0.5 cm downward. The nasal septal mucosal flap was dissected along the subperiosteum with a mucosal stripper, and was turned to the inferior nasal meatus. The nasal septum was fractured to the opposite side to expose the entire sphenoid rostrum. This rescue flap preserves the entire mucosal pedicle of the nasoseptal flap in cases of intraoperative CSF leakage.

### Endoscopic transnasal hemostasis

The 24 patients with delayed epistaxis were treated with emergent endoscopic transnasal hemostasis in our department. All patients were tested for hemoglobin, platelet count and coagulation time in emergency department, and severe coagulation dysfunction was excluded before operation. Hemostatic medication (hemocoagulase) was given and blood pressure was monitored and controlled. Psychological counseling was provided to alleviate patient anxiety. The initial operation video was reviewed to ascertain intraoperative bleeding and exposed arteries. Hemostasis surgery was performed using a 0° endoscope, exploring the bleeding-side nasal cavity. If bleeding was minimal or stopped, the nasal cavity was carefully explored following the principles of shallow-to-deep and top-to-bottom; to clear blood clots and inflammatory edema pale nasal mucosa. The focus of exploration was on bilateral posterior nasal septal artery (PNSA) and incision end of the nasal septal mucosal flap. If there was active bleeding, it was necessary to quickly clear nasal blood clots, explore the responsible artery, control the bleeding point, and then carefully explore other parts of the nasal cavity. If necessary, the sphenopalatine artery or the trunk of posterior nasal septal artery could be found and electrocoagulated at the root of the middle turbinate. After hemostasis was confirmed, absorbable hemostatic gauze and gelatin sponges were used to cover the bleeding site. Hemostasis, infection prevention and supportive treatment were given routinely after surgery. Blood pressure, heart rate, oxygen saturation, red blood cell count and hemoglobin content were monitored 24 h after surgery. Systemic antibiotics were applied for 3 days after surgery. Local antibiotics and menthol nasal drops were administered for 3 weeks. Nasal cavity clearance was performed 1–2 weeks later.

### Statistical analysis

All data were statistically analyzed using SPSS 22.0 software. Continuous variables are presented as mean ± SD, and variables were compared using the unpaired t-test when data were normally distributed or by Wilcoxon rank-sum (Mann-Whitney) test when variables were not normally distributed. Categorical variables are presented as frequencies (%), and intergroup comparisons were analyzed using chi-square tests or Fisher’s exact tests. Binary logistic regression analysis was used to calculate odds ratios (ORs) and 95% confidence intervals (CIs). A two-tailed *P* value < 0.05 was considered to indicate statistical significance.

## Results

### Patient demographics and clinical characteristics

Among the 862 patients who underwent ETPTR, 24 experienced delayed epistaxis, with an incidence rate of 2.78%. Among these patients, there were 14 males and 10 females, with ages ranging from 21 to 67 years (mean age: 47.88 ± 11.59 years). The onset time of delayed epistaxis ranged from 7 to 40 days after surgery, with an average of 20.71 ± 7.39 days and a median of 21 days. A total of 50.0% (12/24) of the bleeding sources involved the posterior nasal septal artery and its branches, 33.3% (8/24) involved multiple inflammatory mucosal bleeding without clear responsible blood vessels, 12.5% (3/24) involved the sphenopalatine artery trunk, and 4.2% (1/24) involved the sphenoid sinus bone. Among the 12 patients who experienced bleeding from the PNSA, 7 had bleeding from the ipsilateral nasal cavity’s PNSA, 3 from the contralateral PNSA, and 2 from the branch of the PNSA at the incision end of the nasal septal mucosal flap. All 24 patients were successfully treated, and no recurrence of epistaxis or other complications occurred.

### Univariate analysis

Our results showed that among patients with delayed epistaxis, the incidence of hypertension was 50% (12/24), which was significantly greater than the incidence of hypertension in patients without delayed epistaxis (26.61%, 223/838) (*p* = 0.01). The incidence of nasal septum deviation was significantly greater in patients with delayed epistaxis (54.17%, 13/24) than in those without delayed epistaxis (16.95%, 142/838) (*p* < 0.01). Similarly, the incidence of chronic rhinosinusitis was significantly greater in patients with delayed epistaxis (41.67%, 10/24) than in those without delayed epistaxis (12.89%, 108/838) (*p* < 0.01). When classified by pituitary tumor subtype, 11 (1.77%) of the 621 patients with nonfunctional pituitary tumors, 10 (6.06%) of the 155 patients with growth hormone-type pituitary tumors, 1 (2.38%) of the 42 patients with corticotropic hormone-type pituitary tumors, and 2 (5.88%) of the 34 patients with prolactin-type pituitary tumors experienced delayed epistaxis. Compared to other tumor subtypes, patients with growth hormone-secreting pituitary tumors had a significantly greater incidence of delayed epistaxis (*p* < 0.01). In summary, a history of hypertension, a deviated nasal septum, chronic rhinosinusitis, and a growth hormone-secreting pituitary tumor subtype were identified as high-risk factors for delayed epistaxis after ETPTR. In addition, sex, age, history of diabetes, tumor size, tumor invasion status, and operation time were not associated with delayed epistaxis. (Table 1)

**Table 1:** Demographic Characteristics

Factor	No epistaxis (*n* = 838)	Epistaxis (*n* = 24)	*p* value
Sex			0.35
Male	407 (48.57%)	14 (58.33%)	
Female	431 (51.43%)	10 ()41.67%	
Age (y)	43.29 ± 14.33	47.88 ± 11.59	0.2
History of hypertension			0.01
Yes	223 (26.61%)	12 (50%)	
No	615 (73.39%)	12 (50%)	
History of diabetes			0.94
Yes	83 (9.9%)	3 (12.5%)	
No	755 (90.1%)	21 (87.5%)	
Chronic rhinosinusitis			< 0.01
Yes	108 (12.89%)	10 (41.67%)	
No	730 (87.11%)	14 (58.33%)	
Subtype			
NF	610 (72.79%)	11 (45.83%)	< 0.01
GH	155 (18.5%)	10 (41.67%)	< 0.01
PRL	32 (3.83%)	2 (8.33%)	0.56
ACTH	41 (4.89%)	1 (4.17%)	0.75
Tumor volume (cm^3^)	9.13 ± 5.46	7.56 ± 8.10	0.09
Invasion			0.39
Yes	204 (24.34%)	4 (16.67%)	
No	634 (75.66%)	20 (83.33%)	
Nasal septal deviation			< 0.01
Yes	142 (16.95%)	13 (54.17%)	
No	696 (83.05%)	11 (45.83%)	
Operation time	178.23 ± 47	163.25 ± 40.55	0.06

ACTH, adrenocorticotropic hormone secreting pituitary tumor; GH, growth hormone secreting pituitary tumor; PRL, prolactin secreting pituitary tumor; NF, Non-functioning pituitary tumor.

### Binary logistic regression analysis

To further elucidate the relationship between the above factors and delayed epistaxis after ETPTR, we performed binary logistic regression analysis, with delayed epistaxis as the dependent variable and all the aforementioned factors as independent variables (Model 1 in Table 2). In order to minimize the bias, we added multivariate logistic model which adjusted for sex, age, course of disease, history of diabetes, smoke and operation time (Model 2 in Table 2). In the adjusted multivariate logistic model, Growth hormone-secreting pituitary tumor subtype (OR = 2.954, 95% CI: 1.139 ~ 7.661, *P* = 0.026), deviated nasal septum (OR = 5.438, 95% CI: 2.091 ~ 14.142, *P* = 0.001), chronic rhinosinusitis (OR = 2.736, 95% CI: 1.027 ~ 7.285, *P* = 0.044) and history of hypertension (OR = 3.089, 95% CI: 1.205 ~ 7.915, *P* = 0.019)were identified as independent risk factors for delayed epistaxis after ETPTR. (Table 2)

**Table 2:** Risk factors for delayed epistaxis analyzed by binary logistic regression

	Model 1					Model 2			
Factor	β	**OR**	95% CI	*p* values		β	**OR**	95% CI	*p* values
**GH subtype**	1.035	2.814	1.118 ~ 7.083	0.028^*^		1.083	2.954	1.139 ~ 7.661	0.026^*^
Tumor volume	-0.062	0.94	0.867 ~ 1.019	0.131		-0.071	0.931	0.856 ~ 1.014	0.1
Invasion	-0.29	0.748	0.241 ~ 2.323	0.615		-0.341	0.711	0.22 ~ 2.295	0.569
**Nasal septal deviation**	1.596	4.934	1.993 ~ 12.215	0.001^*^		1.693	5.438	2.091 ~ 14.142	0.001^*^
**Chronic rhinosinusitis**	1.028	2.795	1.09 ~ 7.165	0.032^*^		1.006	2.736	1.027 ~ 7.285	0.044^*^
**History of hypertension**	0.949	2.582	1.082 ~ 6.16	0.032^*^		1.128	3.089	1.205 ~ 7.915	0.019^*^

Model 1 was an univarilate logistic model; Model 2 was adjusted for sex, age, course of disease, history of diabetes, smoke and operation time. GH, growth hormone secreting pituitary tumor; *, significant statistical difference.

### Follow-up

All patients were followed up for more than 6 months to assess postoperative recovery, including recurrent epistaxis, nasal discomfort, basic disease control and psychological status. All 24 patients achieved satisfactory recovery with no recurrence of bleeding during the follow-up period.

## Discussion

### Clinical characteristics

Delayed epistaxis, a rare postoperative complication of ETPTR, has not received sufficient attention for a long time. Previous reports have shown that the incidence of epistaxis after endoscopic transnasal surgery ranges from 1.1 to 3.3%. De Los Reyes reported 18 cases (3.3%) of epistaxis among 551 endoscopic surgeries [3]. Thompson reported that epistaxis occurred in 10 (3.0%) of 330 patients after endoscopic sinus surgery [6]. Stankiewicz retrospectively studied 3402 endoscopic surgery cases and reported an epistaxis incidence rate of 1.1% [7]. The incidence of delayed epistaxis in our study was 2.78%, which is consistent with previous reports.

Although with low incidence, delayed epistaxis tends to cause serious consequences, such as aspiration and hemorrhagic shock. Because the bleeding source is often located in the posterior part of the nasal cavity, conventional nasal packing hemostasis is often ineffective. Nasal mucosal artery injury is the direct cause of delayed epistaxis. The sphenopalatine artery is the main blood supply to the nasal mucosa and is divided into the lateral posterior nasal artery and PNSA. The PNSA crosses the anterior inferior part of the sphenoid sinus. It can be damaged during mucosal flap manipulation and high-speed drilling operations. Therefore, the PNSA is considered to be the primary vessel responsible for postoperative epistaxis [6]. In our study, 50.0% of epistaxis bleeding sources were from the PNSA and its branches. Therefore, we recommend focusing on exploring the bilateral PNSA trunk and the incision end of the mucosal flap during endoscopic transnasal hemostasis. By reviewing the video of the patients’ initial ETPTR operation, we found that among the 24 patients with delayed epistaxis, only 2 had clearly responsible arterial bleeding. Moreover, during the second hemostasis surgery, mucosal inflammation around the responsible artery is commonly observed. Therefore, we infer that most arterial injuries leading to delayed epistaxis occur postoperatively. The possible reasons could be as follows: (1) Intraoperative damage to the mucosa adjacent to artery, which has not fully healed postoperatively. (2) Intraoperative nasal packing compresses the nasal mucosa, leading to mucosal ulceration, edema and necrosis. (3) Local inflammatory reactions invade the arteries.

Previous studies have not reported the typical onset time of delayed epistaxis after ETPTR. Our study revealed that delayed epistaxis mainly occurred at 3 weeks post operation (average of 20.71 ± 7.39 days and median of 21 days). This time characteristic does not have a clear theoretical basis, but we believe that it is related to the repair process of the nasal mucosa after endoscopic nasal surgery. Postoperative repair of the nasal mucosa after endoscopic surgery can be divided into three stages. The first stage, 1–2 weeks after surgery, is a period of nasal cavity cleaning and drying, during which mucosal edema gradually decreases, secretions decrease, and blood scabbing gradually disappears, resulting in a cleaner surgical cavity. The second stage, 3–10 weeks after surgery, is the competition stage of mucosal repair. During this stage, edema reappears, and vesicles, polyps, and granulation appear at the site of mucosal defects. Moreover, mucosal regeneration and epithelialization also occur. Mucosal regeneration and lesions occur simultaneously and exhibit competitive growth. The third stage occurs 10 weeks after surgery when epithelialization of the cavity is complete [8, 9]. The first 1–2 weeks after surgery are a critical period for mucosal repair. If certain factors cause premature shedding of blood scabs or excessive local inflammatory reactions, mucosal repair may be inadequate, leading to the reappearance of edema and inflammation in the third week, which can cause delayed epistaxis.

### Risk factors

Delayed epistaxis after ETPTR may be related to various factors, including patient-specific factors, surgery-related factors, and postoperative management factors. The analysis of relevant risk factors is essential for preventing delayed epistaxis. Our results showed that growth hormone, pituitary tumor subtype, nasal septum deviation, chronic rhinosinusitis and history of hypertension are independent risk factors for delayed epistaxis. The turbinate, nasal septum and sphenoid sinus bone are often hyperplastic and hard in patients with GH pituitary adenomas. It is difficult to shift or remove these structures, which increases the possibility of mucosal vascular injury. Moreover, due to the excessive secretion of growth hormone, the nasal mucosa is hypertrophic and edematous, and the blood supply of redundant tissues is rich, which increases the possibility of rebleeding of small blood vessels after nasal compression is relieved [10]. For patients with nasal septum deviation, the deviant bone of the nasal septum deviation needs to be removed. The exposure and injury of deviant bone easily causes inflammation without mucosal protection. In addition, a higher blood flow impact pressure, thinner mucosa and harder airflow pressure easily cause damage to blood vessels at the deviated nasal septum. Therefore, nasal septum deviation should be evaluated by nasal sinus CT before surgery, and the more spacious nasal cavity side should be chosen as the endoscopic approach. Rhinosinusitis leads to increased nasal mucosal bleeding during surgery, thereby reducing the visibility of the surgical field and increasing the risk of mucosal vascular injury. Inflammation can also cause submucosal fibrosis, which may make it more difficult to protect against submucosal dissection of the mucosal flap and the PNSA [4]. Moreover, the preoperative inflammatory condition of the nasal cavity delays postoperative nasal mucosa repair, potentially leading to the invasion of inflammation into blood vessels and subsequent delayed epistaxis. The incidence of delayed epistaxis significantly increases in patients with hypertension because hypertension causes decreased elasticity and increased brittleness of small arteries, increasing the risk of postoperative bleeding. Therefore, stable preoperative blood pressure control is essential to reduce the incidence of delayed epistaxis in hypertensive patients [11]. There was no significant correlation between tumor size, invasion, operation time or delayed epistaxis. Tumor size does not directly represent the difficulty of surgery. Although factors such as invasiveness can lead to prolonged operation time, most surgical manipulations are performed intracranially, and there is no significant increase in nasal injury; therefore, this approach does not significantly increase the possibility of postoperative epistaxis. Patients who received improper nasal packing had significantly poorer mucosal repair at 3–4 weeks postoperatively [12]. we recommend nasal packing with iodoform gauze (within 3 days), because iodoform gauze can effectively act as an anti-inflammatory and drainage, prevent postoperative nasal inflammation and adhesion, and thus prevent delayed epistaxis. We do not recommend nasal packing with Merocel because of its lack of anti-inflammatory effects.

### Treatment and prevention

Treatment for delayed epistaxis includes endoscopic hemostasis, nasal packing and endovascular embolization [13]. Once delayed epistaxis occurs, nasal packing should be performed as early as possible in the emergency department. Considering that the source of epistaxis is mostly located in the posterior part of the nasal cavity, the failure rate of nasal packing hemostasis is relatively high. In addition, emergent nasal packing may cause mucosal compression and inflammation, leading to rebleeding. Therefore, we recommend emergent endoscopic hemostasis for delayed epistaxis, full exploration of the responsible blood vessels or electrocoagulation of the main trunk of the PNSA or sphenopalatine artery if necessary. For fatal epistaxis caused by internal carotid artery injury, emergent endovascular embolization is needed. Prevention of delayed epistaxis should be implemented throughout the perioperative period. Preoperative imaging data of nasal sinuses, including whether there is rhinosinusitis, turbinate hypertrophy, nasal septum deviation, etc., should be evaluated. Preoperative application of glucocorticoids can inhibit nasal mucosal vasodilation and vascular plexus formation caused by rhinosinusitis [14]. During the operation, the nasal mucosa should be protected carefully, and a mucosal flap should be formed in reasonable parts and of a reasonable size to avoid injury to the main trunk of the sphenopalatine artery and PNSA. Favier et al. [15] developed a subperichondrial transseptal approach, which could entirely preserve the mucosa of the anterior wall of the sphenoid sinus, thus minimizes the risk of rhinogenic infections. There is also no risk of injury to the posterior nasal artery, which decreases the risk of postoperative epistaxis. At the end of the operation, the nasal mucosa should be fully explored, and the necrotic tissue, residual blood clots and bone slices should be carefully cleared. Nasal debridement was performed 1–2 weeks after surgery. A nasal douche with normal saline helps to increase local blood circulation and promote mucociliary clearance and mucosal epithelialization. Nonhalogenated glucocorticoid steroid nasal sprays have local antiallergic, anti-inflammatory and antiedema effects and can effectively control the growth of vesicles and small polyps [8]. Because the occurrence of delayed epistaxis mainly occurs 3 weeks postoperatively, we recommend that preventive measures for risk factors be maintained for at least 3 weeks.

### Limitations

There are also some limitations to this study because of its retrospective nature. First, the major limitation of our study is the small sample size because of the low incidence of delayed epistaxis. The results of the statistical analysis may lack statistical power. Second, as a single-center study, our clinical recommendations are interim and restricted, and more evidence from large-scale and multicenter studies is required to establish standardized treatment guidelines. Finally, this study excluded patients who experienced minor epistaxis, which spontaneously stopped outside the hospital and did not require surgical treatment. The clinical information of these patients was not included in the statistical analysis, which may have caused bias in the clinical characteristics of patients with delayed epistaxis. Despite the limitations mentioned above, this is the first study exploring the clinical characteristics and risk factors for delayed epistaxis after ETPTR, which can guide neurosurgeons and patients and families in discussions centered on optimal perioperative surveillance based on individualized risk factors.

## Conclusion

The PNSA is the primary vessel responsible for delayed epistaxis. Conventional nasal packing hemostasis is often ineffective, endoscopic transnasal hemostasis is recommended as the preferred treatment, and exploration of the bilateral PNSA trunk and the incision end of the mucosal flap should be focused on. The occurrence of delayed epistaxis mainly occurred 3 weeks postoperatively. A history of hypertension, nasal septum deviation, chronic rhinosinusitis, and growth hormone pituitary tumor subtype are independent risk factors for delayed epistaxis. Preventive measures for risk factors should be maintained at least 3 weeks after surgery.

## Data Availability

No datasets were generated or analysed during the current study.

## References

[1] Castaño-Leon AM, Paredes I, Munarriz PM (2020). Endoscopic Transnasal Trans-Sphenoidal Approach for Pituitary adenomas: a comparison to the Microscopic Approach Cohort by Propensity Score Analysis.[J]. Neurosurgery.

[2] Nishioka H, Ohno S, Ikeda Y (2007). Delayed massive epistaxis following endonasal transsphenoidal surgery[J]. Acta Neurochir (Wien).

[3] De Los RK, Gross BA, Frerichs KU (2015). Incidence, risk factors and management of severe post-transsphenoidal epistaxis[J]. J Clin Neurosci.

[4] Kam J, Ahmad A, Williams A (2019). Postoperative epistaxis and sphenoid sinus ostial stenosis after posterior septal branch injury during sphenoidotomy[J]. Int Forum Allergy Rhinology.

[5] Rawal RB, Kimple AJ, Dugar DR (2012). Minimizing morbidity in endoscopic pituitary surgery: outcomes of the novel nasoseptal rescue flap technique[J]. Otolaryngol Head Neck Surg.

[6] Thompson CF, Wang MB, Kim BJ (2012). Incidence and management of epistaxis after endoscopic skull base surgery[J]. ORL J Otorhinolaryngol Relat Spec.

[7] Stankiewicz JA, Lal D, Connor M (2011). Complications in endoscopic sinus surgery for chronic rhinosinusitis: a 25-year experience[J]. Laryngoscope.

[8] Xu G, Li Y, Xie M (1999). [Staging of mucous membrane outcome in operative cavity after functional endoscopic sinus surgery][J]. Zhonghua Er Bi Yan Hou Ke Za Zhi.

[9] Xu G, Jiang H, Li H (2008). Stages of nasal mucosal transitional course after functional endoscopic sinus surgery and their clinical indications[J]. ORL J Otorhinolaryngol Relat Spec.

[10] Zada G, Cavallo LM, Esposito F (2010). Transsphenoidal surgery in patients with acromegaly: operative strategies for overcoming technically challenging anatomical variations[J]. Neurosurg Focus.

[11] Kikidis D, Tsioufis K, Papanikolaou V (2014). Is epistaxis associated with arterial hypertension? A systematic review of the literature[J]. Eur Arch Otorhinolaryngol.

[12] Wang YP, Wang MC, Chen YC (2011). The effects of Vaseline gauze strip, Merocel, and Nasopore on the formation of synechiae and excessive granulation tissue in the middle meatus and the incidence of major postoperative bleeding after endoscopic sinus surgery[J]. J Chin Med Assoc.

[13] Alzhrani G, Sivakumar W, Park MS (2018). Delayed complications after Transsphenoidal surgery for pituitary Adenomas[J]. World Neurosurg.

[14] Mullol J, Alobid I (2011). Combined oral and intranasal corticosteroid therapy: an advance in the management of nasal polyposis?[J]. Ann Intern Med.

[15] Favier V, Le Corre M, Segnarbieux F, et al. Endoscopic subperichondrial transseptal transsphenoidal approach is safe and efficient for non-extended pituitary surgery[J]. Volume 277. European Archives of Oto-Rhino-Laryngology; 2020. pp. 1079–87. 4. 10.1007/s00405-020-05790-6 31960129

